# Internal fixation of a fractured cranial articular process of the sixth cervical vertebra by means of computer‐assisted surgery in a Warmblood gelding

**DOI:** 10.1111/vsu.14273

**Published:** 2025-05-23

**Authors:** Melanie J. Käfer‐Karrer, Mathieu de Preux, Elke Van der Vekens, Larissa I. Mattei, Jovana Kuhlmann, Micaël D. Klopfenstein Bregger, Jeremiah T. Easley, Christoph Koch

**Affiliations:** ^1^ Division of Equine Surgery, Department of Clinical Veterinary Medicine, Vetsuisse Faculty University of Bern Bern Switzerland; ^2^ Division of Clinical Radiology, Department of Clinical Veterinary Medicine, Vetsuisse Faculty University of Bern Bern Switzerland; ^3^ Section of Anesthesiology and Pain Therapy, Department of Clinical Veterinary Medicine, Vetsuisse Faculty University of Bern Bern Switzerland; ^4^ Preclinical Surgical Research Laboratory, Department of Clinical Sciences, Translational Medicine Institute Colorado State University Fort Collins Colorado USA

## Abstract

**Objectives:**

To describe the repair of a fractured cranial articular process (AP) of the sixth cervical vertebra (C6) with cortex screws placed in lag fashion using computer‐assisted surgery.

**Animal:**

A 12‐year‐old Swiss Warmblood gelding.

**Study design:**

Case report.

**Methods:**

The gelding was presented with neck pain and ataxia after a fall. A fracture of the right cranial AP of C6 was identified on radiographs. The fracture was repaired by internal fixation with two 4.5 mm cortex screws of 58 and 48 mm length placed in lag fashion using computer‐assisted surgery.

**Results:**

During general anesthesia, it was suspected that the gelding developed brain edema that prolonged the recovery process. Postoperatively, the horse showed transient ataxia and myopathy of the left triceps muscle. Except for focal muscular atrophy of the right supraspinatus muscle, all complications resolved. Radiographs confirmed healing of the fracture with minimal callus formation. One year after surgery, the gelding had returned to ridden exercise and was performing well.

**Conclusion:**

Computer‐assisted surgery facilitates lag screw fixation of AP fractures and makes this a potential alternative to intervertebral body fusion, which is recommended for this type of fracture to avoid excessive callus formation and subsequent spinal cord or cervical spinal nerve compression.

## 1 INTRODUCTION

Fractures of equine cervical vertebrae result from hyperextension, hyperflexion, or lateral bending injuries and are usually sustained during falls.^1^ While injuries in foals commonly result in fractures of the cranial cervical vertebrae (C2–C4), in adults, the mid‐cervical region (C3–C6) is more prone to traumatic injury.^1^ Articular process (AP) fractures are the second most common type of cervical vertebral fractures, as the bony pedicles of the AP represent the weakest point of the vertebrae.^1^, ^2^, ^3^

Clinical signs are highly variable and depend on the degree of spinal cord compression. Not all cervical fractures lead to instability and, therefore, neurologic impairment. Clinical signs may be absent or range from abnormal neck position and pain to neurologic deficits, including transient or persistent ataxia of variable severity.^4^ A neurologic and radiographic work‐up is mandatory to assess possible spinal cord compression. In cases with mild transient ataxia and minimally displaced fractures, conservative treatment is a viable option. However, the formation of exuberant callus consequential to secondary fracture healing may result in spinal cord or cervical spinal nerve compression due to foraminal stenosis, potentially necessitating later surgical intervention.^1^, ^4^

Descriptions of the surgical management of AP fractures are thus far limited to intervertebral body fusion. Specifically for AP fractures, ventral cervical stabilization using a Bagby basket^5^ or a Kerf‐cut cylinder^1^ have been described. Placement of cortex screws in lag fashion requires meticulous surgical precision, as the AP is a thin osseous structure bordered by critical neurovascular structures, leaving little room for error.^3^, ^5^ Stabilization of a luxated articular process joint (APJ) via a direct approach using cortex screws placed in lag fashion has been reported.^6^

Computer‐assisted surgery (CAS) has proven helpful for various indications in equine surgery.^7^, ^8^, ^9^ Preoperative multiplanar imaging and virtual modeling enable detailed surgical planning. Intraoperatively, CAS allows for precise real‐time orientation, facilitating minimally invasive approaches and optimizing drilling trajectory, particularly in anatomically challenging areas.^9^ These features make CAS an ideal supportive technique for spinal surgery.^10^ This is the first report describing the application of CAS for the repair of a fractured AP of C6 in a horse using internal fixation with cortex screws placed in lag fashion.

## 2 MATERIALS AND METHODS

### 2.1 Case history

A 12‐year‐old Swiss Warmblood gelding, used for mid‐level dressage, was presented at the ISME Equine Clinic of Bern, Switzerland, after falling on his left side and being recumbent for minutes before getting up. Once back on his feet, the horse remained stiff and was reluctant to move.

### 2.2 Clinical findings

Upon arrival, the gelding appeared painful, moving hesitantly with shortened anterior phases of the stride and clear signs of hindlimb ataxia (grade 2 on the modified Mayhew scale^11^). The neurologic examination was otherwise unremarkable. Neck mobility was reduced, and palpation of the mid‐cervical region was painful bilaterally. Left–right laterolateral, left 45° ventral‐right dorsal oblique and right 45° ventral‐left dorsal oblique radiographic projections of the cervical spine were made, revealing a transverse radiolucent line in one of the APs of the right APJ at the level C5/6. The gelding was medicated with flunixin meglumine (1.1 mg/kg IV once daily) and dexamethasone (0.05 mg/kg IV once at admission) and showed rapid improvement. The hindlimb ataxia resolved the following day, and the horse was sent home after two days with instructions for stall confinement.

**FIGURE 1:**
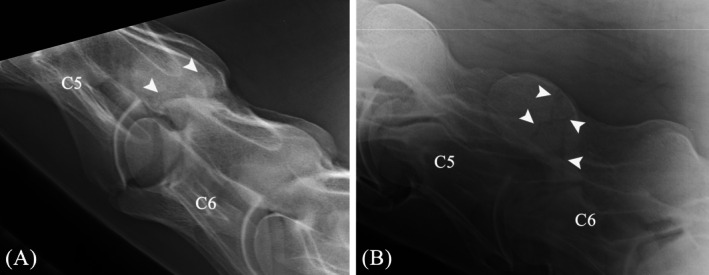
Preoperative radiographs taken five weeks after the incident. (A) Laterolateral projection. (B) Right 45° ventral‐left dorsal oblique projection, showing the fracture line in the right cranial AP of C6 (arrowheads).

The gelding was presented for a recheck five weeks after the incident. No lameness or ataxia was observed, but neck mobility was still reduced, and the gelding remained painful to palpation in the cervical region. Radiographs were repeated and confirmed the presence of a fracture line of the right cranial AP of C6 with extension into the vertebral arch of C6 (Figure 1). Given the concerns about increasing fracture displacement and exuberant callus formation leading to spinal cord compression or foraminal stenosis, internal fracture fixation was elected to minimize the risk of developing athletic restrictions due to ataxia or cervical nerve compression. Surgery was performed eight weeks after the accident.

### 2.3 Preoperative diagnostic imaging

Prior to induction of general anesthesia, the horse was premedicated with benzylpenicillin sodium (30 000 IU/kg IM), gentamycin (6.5 mg/kg IV), flunixine meglumine (1.1 mg/kg IV), and acepromazine (0.3 mg/kg IM). After sedation with romifidine (0.06 mg/kg IV) and levomethadone (0.05 mg/kg IV), an Equiporter Sling (Dr. Fritz Endoscopes GmbH, Buchheim‐Tuttlingen, Germany) was placed on the gelding. General anesthesia was induced with ketamine (2.5 mg/kg IV) combined with diazepam (0.05 mg/kg IV). The horse was positioned in left lateral recumbency on the operating table and the head and neck were placed in an extended position on a malleable vacuum pad on a carbon fiber table (Opera Swing; General Medicale Merate SPA, Seriate, Italy).

**FIGURE 2:**
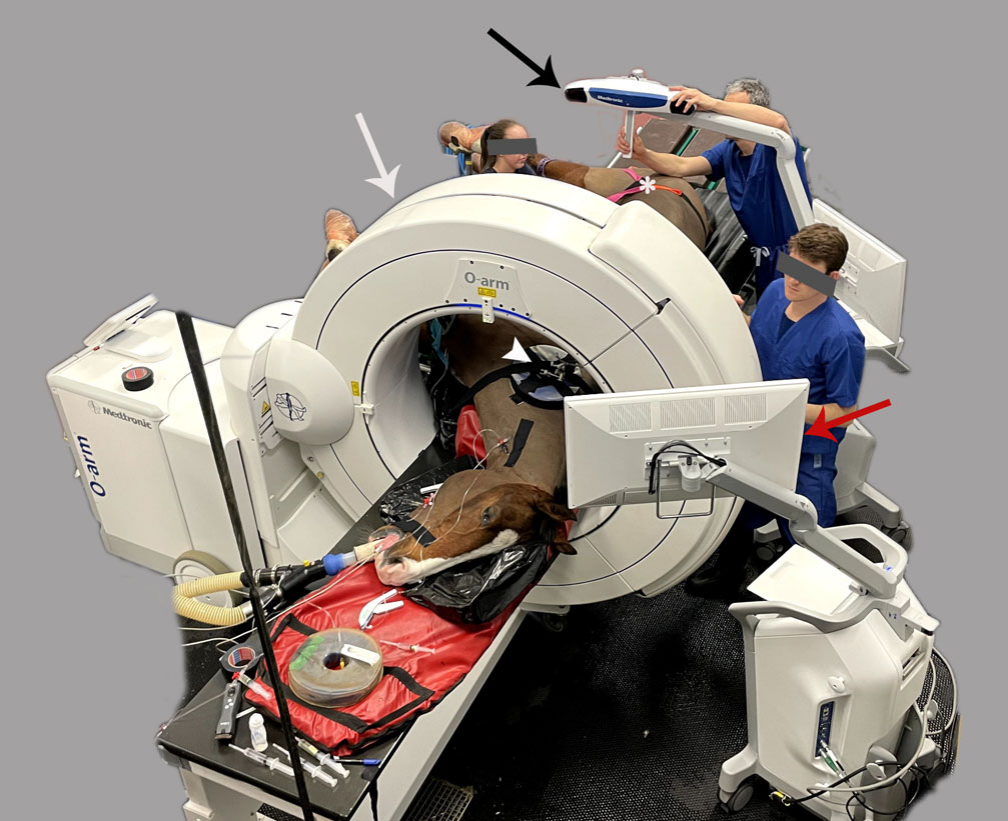
Overview of the setup for computer‐assisted surgery immediately before image acquisition. The O‐arm (white arrow) coupled with the StealthStationS8 (red arrow), and the beacon of the camera (black arrow) oriented to simultaneously detect both patient and gantry tracker are shown. The patient tracker (white arrowhead) is taped to the right lateral aspect of the neck. The Equiporter Sling (white asterisk) is already placed on the horse.

**FIGURE 3:**
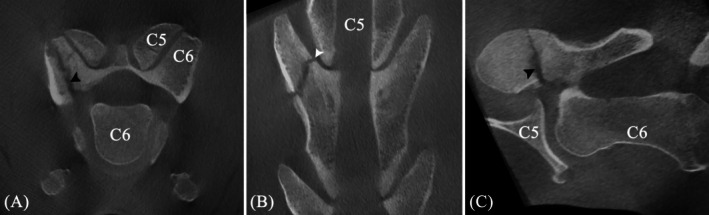
Preoperative cone beam computed tomography (CBCT) images centered on the fifth (C5) and sixth (C6) cervical vertebrae. (A) Transverse, (B) dorsal and (C) sagittal multiplanar reconstructions of the right cranial articular process (AP) of C6 show a wide, ill‐defined fracture line (black arrowheads) with a craniodorsomedial to caudoventrolateral orientation, transverse to the long axis of the AP. An additional small, but non‐displaced triangular fragment is visible at the dorsal aspect of the AP, immediately caudal to the main fracture line. Both fracture lines are interrupting the articular surface (white arrowhead). Mild irregular periosteal reaction was present in the main fracture fragments.

A cone beam CT (CBCT) unit (O‐arm; Medtronic, Louisville, Colorado) coupled with a surgical navigation system (StealthStationS8; Medtronic) was used for all pre‐ and intraoperative imaging. For initial orientation and to guide patient tracker fixation on the transverse process of C6, the patient tracker (passive orthopedic reference frame; Medtronic) was taped to the right lateral aspect of the neck fixed in a foam platform (Figure 2). A first preoperative CBCT scan was acquired and automatically transferred to the StealthStationS8. Images revealed a 3 mm wide fracture gap in the right cranial AP of C6. The fracture coursed in a craniodorsomedial to caudoventrolateral direction transverse to the long axis of the AP. Due to minimal fracture displacement, the joint space of the involved APJ appeared narrowed at its caudal aspect (Figure 3). A small, non‐displaced, triangular fragment was present at the dorsal aspect of the AP, immediately caudal to the main fracture, creating mild comminution. Ventrally, the main fracture extended along the cranial border of the lateral lamina to the transverse foramen. Subtle irregular periosteal reaction was present, resulting in a slight intervertebral foramen narrowing. The contralateral APJ was unremarkable.

Using the navigated pointer, the outline of the surgical field and the spatial orientation of the right transverse process of C6 were determined for subsequent placement of the anchoring Schanz pins.

### 2.4 Surgical approach

**FIGURE 4:**
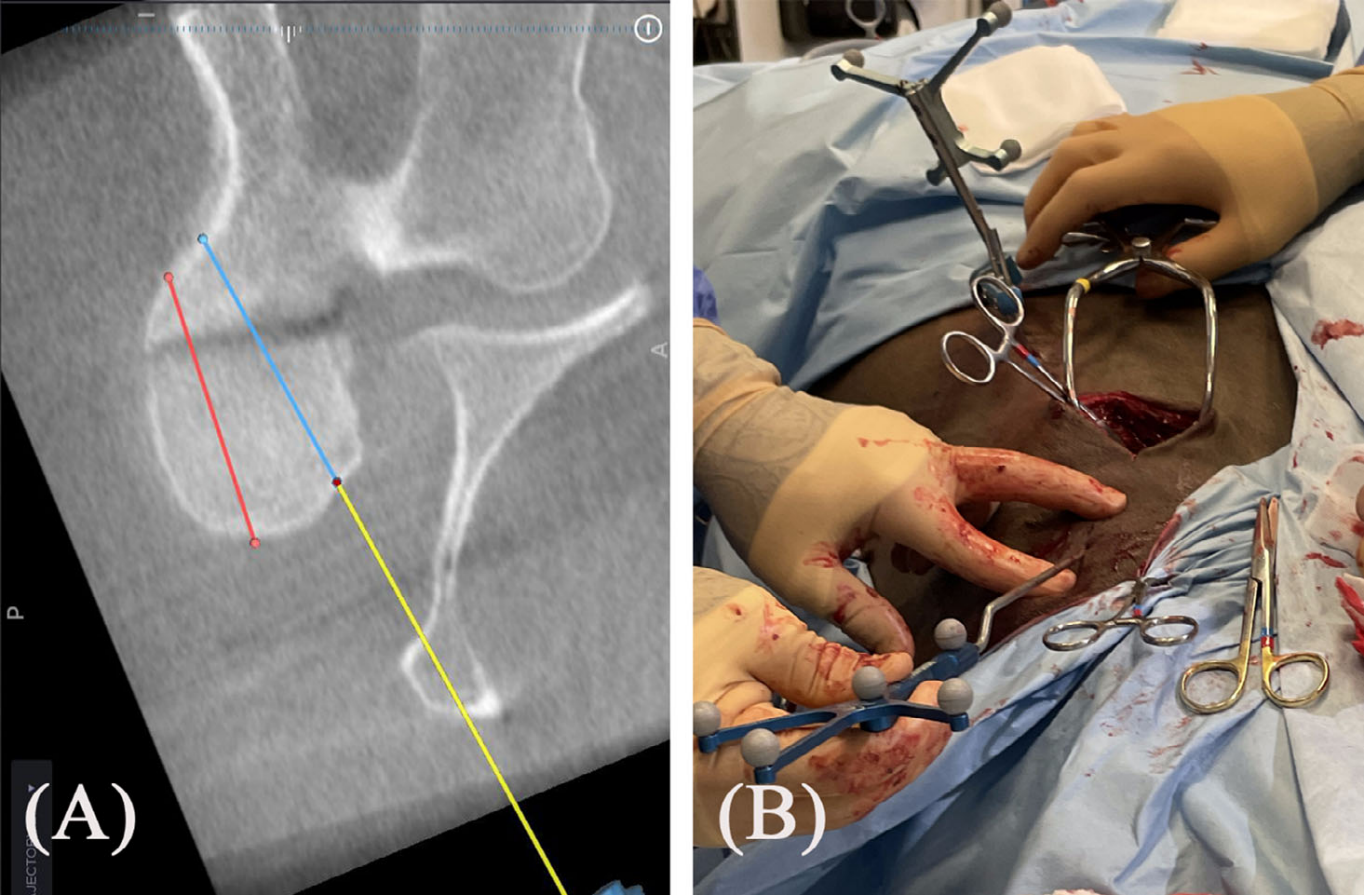
Surgical planning. (A) Screenshot from the navigation software of the StealthStationS8 showing the plan for the placement of two cortex screws (red and blue line) and the trajectory for the first plan (yellow line). (B) The navigated pointer is used to establish the location for the incision to line up the instruments with the trajectory.

After aseptic preparation and draping, two self‐tapping threaded 3.2 mm Schanz pins were placed through stab incisions in the right transverse process of C6 to anchor a sterile patient tracker to C6. A second preoperative CBCT scan was acquired. A surgical plan for the placement of two 4.5 mm cortex screws in lag fashion across the fractured AP in a cranial to caudal direction was drawn on the acquired CBCT data set using the navigation software of the StealthStationS8 (Spine and Trauma; Medtronic) (Figure 4A).

**FIGURE 5:**
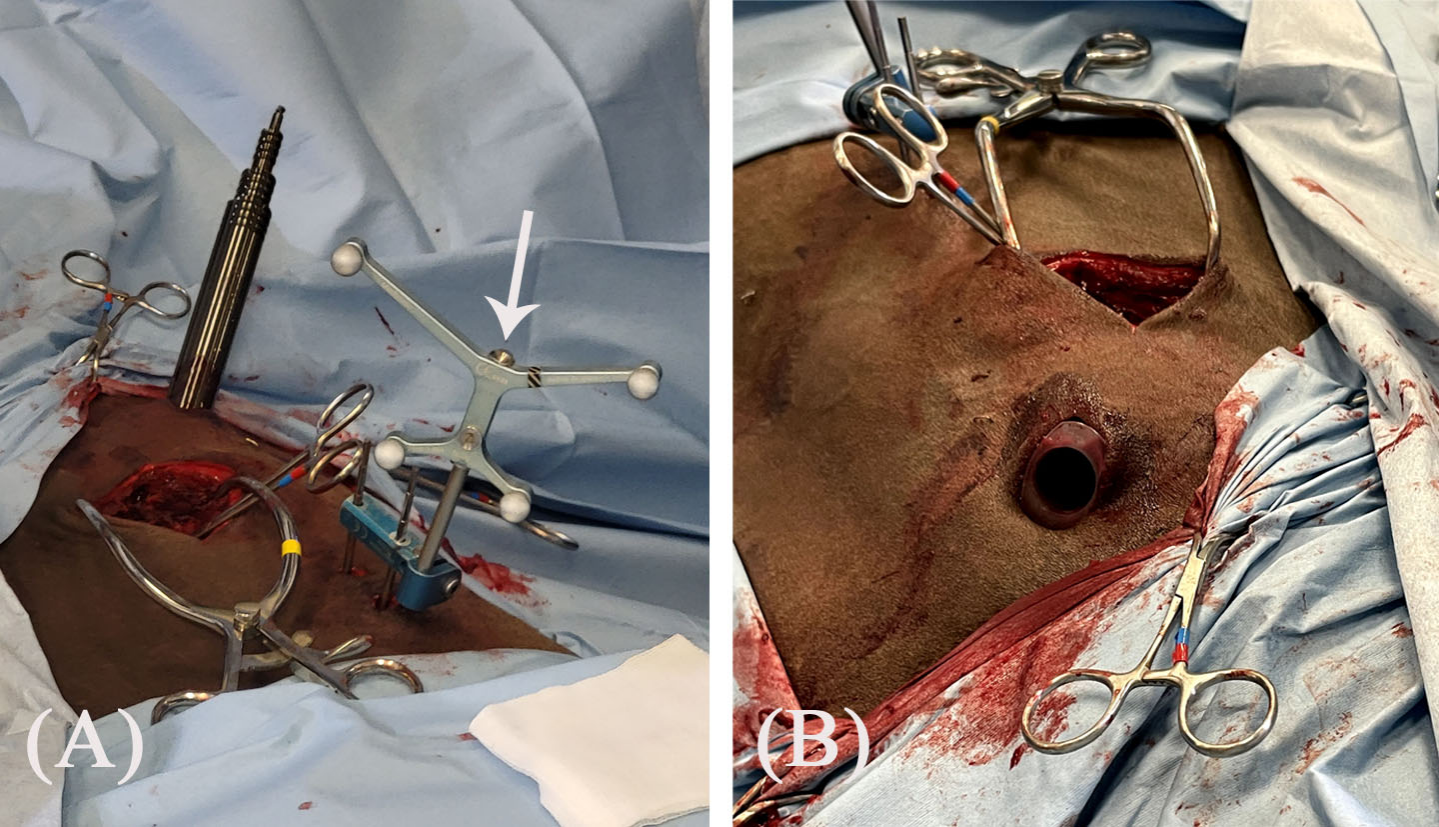
Intraoperative photographs. (A) Caudal incision with a Gelpi retractor placed for optimal visualization, and the soft tissue dilator placed in the cranial incision. The patient tracker (arrow) is anchored to the transverse process of C6. Top – cranial, bottom – caudal. (B) The dilator has been replaced by a cut up 35 cc syringe to act as a sleeve. Top – caudal, bottom – cranial.

The battery‐powered surgical drill (Colibri II; DePuy Synthes, West Chester, Pennsylvania) was mounted with a small tracker (SureTrak II clamps and tracker; Medtronic) on the instrument shaft and calibrated.^9^ The appropriate location of the skin incisions was determined using the navigated pointer. An 8 cm skin incision was made directly over the APJ of C5/6, and a combination of sharp and blunt dissection was made through the overlaying muscles to expose the APJ. A second 5 cm skin incision was made approximately 10 cm cranial so that the long axis of the navigated instruments could be aligned with the trajectories of the surgical plan of both screws (Figure 4B). To prepare the cranial access, a tubular soft tissue dilator set (Direct Lateral Dilators; Medtronic) was used. The guidewire of the set was equipped with a small instrument tracker (SureTrak II Universal Tracker; Medtronic) and placed on the cranial aspect of the targeted AP under navigation guidance (Figure 5A). A working channel was created by sequentially inserting the dilators over the guidewire. After removing the stamp and cutting the tip, a 35 mL syringe was introduced over the largest dilator and served as a speculum to establish the cranial surgical approach (Figure 5B).

### 2.5 Screw placement

**FIGURE 6:**
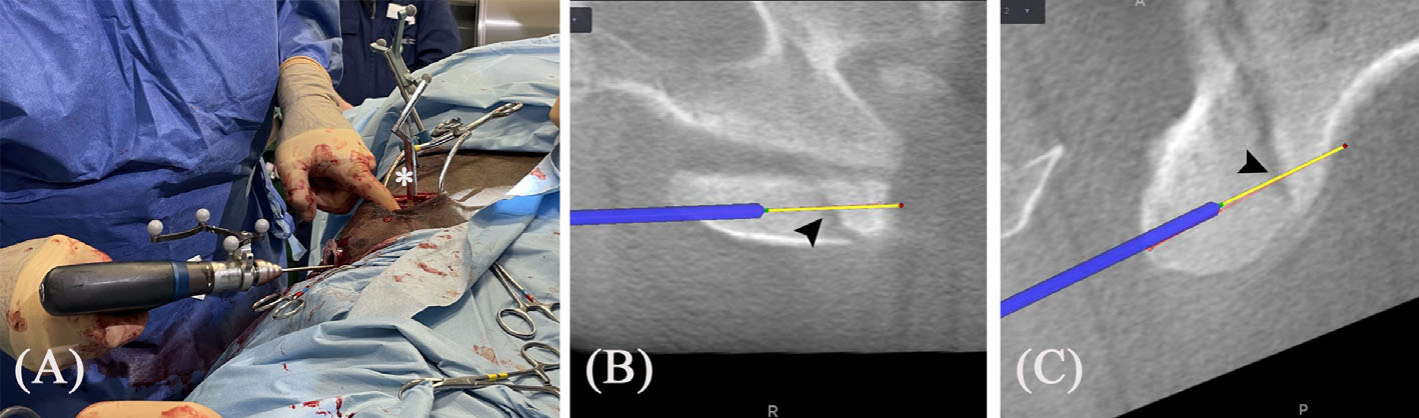
Navigated drilling. (A) The drill sleeve (asterisk) is inserted through the caudal approach, and the drill bit through the cranial approach. (B–C) Corresponding view on the navigation software of the StealthStationS8. Red line (mostly obscured by the path of the drill bit): Surgical plan for screw position. Yellow line: Target aim. Thick blue line: Drill bit. The drill bit has already penetrated the cis cortex but has not reached the fracture line (arrowheads).

**FIGURE 7:**
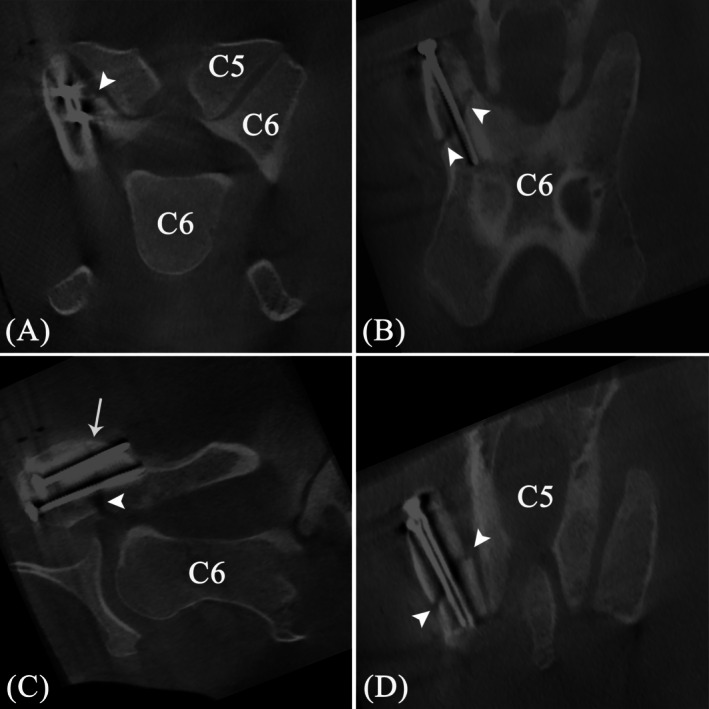
Intraoperative cone beam computed tomography (CBCT) images. (A) Transverse, (B,D) dorsal, and (C) sagittal multiplanar reconstructions of an 8‐week‐old fracture (arrowheads) of the right cranial AP of the sixth cervical vertebra (C6) with the two 4.5 mm cortex screws in situ. Perfect apposition of the fracture fragments was achieved at the level of the dorsal screw (arrowhead, B) and a minimal step was present just dorsal to the ventral screw (D). The small additional triangular fragment at the dorsal aspect remained in place during fracture fixation (arrow, C). Note the beam hardening artifacts present in all images due to the metal screws.

Once both approaches were established and the surgeon was able to readily access the desired entry points of the planned drill tracts, a navigated high‐speed surgical drill (Midas MR8; Medtronic) mounted with a 5 mm round‐head burr was used to create bit seats for both surgical plans in the cranial contour of the AP to prevent slipping of the drill bit on the slanted and narrow cranial aspects of the AP. After preparing the bit seats, the navigated battery‐powered surgical drill was used to prepare the glide holes with a 4.5 mm (195 mm long) drill bit and the thread holes with a 3.2 mm (195 mm long) drill bit for the placement of cortex screws in lag fashion. For each surgical plan, the drill bit was introduced through the syringe speculum, and the drill sleeve was inserted through the direct approach over the AP (Figure 6A). Each hole was drilled under real‐time visual control over the drill orientation and penetration depth provided by the navigation system (Figure 6B,C). The screw lengths were measured on the CBCT scan, and 4.5 mm cortex screws of 58 mm and 48 mm length were inserted after tapping and countersinking. An intraoperative CBCT scan was performed to control adequate placement and length of the inserted screws before removing the patient tracker and anchoring pins (Figure 7). The incisions were closed using absorbable 2‐0 polyfilament suture material (Vicryl; Ethicon, Johnson & Johnson, Raritan, New Jersey) in a simple continuous pattern for the subcutaneous tissue, and nonabsorbable 2‐0 monofilament suture material (Prolene; Ethicon) in a simple interrupted pattern for the skin. Both incisions were covered with sterile gauze and a self‐adhesive foam dressing (Animal Polster; Snögg, Solna, Sweden) and protected with a self‐adhesive polyurethane foil (Opsite Incise Drape; Smith & Nephew, London, UK).

### 2.6 Recovery

Recovery was assisted using a sling system and support frame (Dr. Fritz Endoscopes GmbH) in combination with a balancer (Balancer Type 7261; Kromer GmbH, Gotteneheim, Germany) and head and tail ropes. While the horse was stable during general anesthesia and no complications were noted during the anesthetic period, the gelding initially showed a complete absence of ventilatory drive. An arterial blood gas analysis was performed, showing a PaCO2 of 90 mmHg. All reflexes were absent at that moment, presumably due to brain edema, leading to increased intracranial pressure (ICP). The horse was positioned and maintained in sternal position with the help of the sling and ventilated using a demand valve with 100% oxygen for about 45 min (average six breaths/min). The head was placed on a cushion in an elevated position, and 2 L of hypertonic saline were administered IV to counteract the suspected brain edema. The clinical condition of the gelding improved, and further recovery was uneventful. Total surgery time was 200 min, total anesthesia time was 290 min, and time between the end of anesthesia and standing was 95 min.

### 2.7 Postoperative care

Broad‐spectrum intravenous antimicrobial prophylaxis and anti‐inflammatory medication were continued for another five days postoperatively. The day after surgery, the gelding seemed depressed and painful and showed grade 3 ataxia.^11^ With generalized hyperesthesia, the gelding reacted particularly painful to palpation at the head and neck, and in a well‐circumscribed area over the left scapula, an edematous swelling was noted. Consequently, he was administered dexamethasone (0.1 mg/kg IV) and a multimodal analgesic treatment, including gabapentin (10 mg/kg orally three times daily), paracetamol (20 mg/kg orally twice daily), and ketamine (0.4 mg/kg subcutaneously every 4 h).

In the following days, analgesic treatment was gradually reduced. Four days after surgery, the hindlimb ataxia had improved, but the gelding showed a reduced anterior phase of the stride of his front left at the walk. The swelling over the left triceps muscle remained visible but was less painful on palpation. By day 6 after surgery, the ataxia had improved markedly, and the gelding was discharged seven days postoperatively. No wound‐healing complications, such as seroma formation or incisional drainage, were noted at any time postoperatively. Treatment with flunixine meglumine and gabapentin was continued at home for 5 and 20 days, respectively. The owner was instructed to keep the horse on stall rest with access to a small paddock and to start in‐hand walking two weeks postoperatively twice daily for 10–15 min until the first recheck.

## 3 RESULTS

**FIGURE 8:**
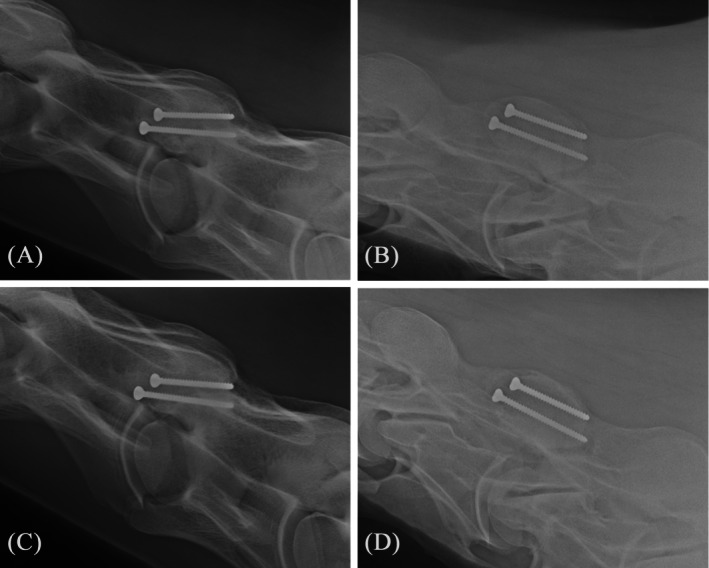
Postoperative radiographs taken (A–B) eight weeks and (C–D) eight months after surgery where the fracture line is not visible anymore. (A/C) Laterolateral projection. (B/D) Right 45° ventral‐left dorsal oblique projection.

The gelding was presented for the first recheck 8 weeks after surgery. A distinct atrophy of the left supraspinatus muscle had developed at this stage. He also showed painful cervical swelling at the level of C5/6 and was reluctant to flex his neck with decreased mobility to the left and right. Very mild ataxia of the hindlimbs was questionably present when turning on small circles (grade 1). Control radiographs showed that the two cortex screws were intact and in place, with progressive fracture healing without exuberant callus formation (Figure 8). The owner was thus instructed to turn the gelding out in the field and start light lunging exercise for another 6 weeks before beginning to ride the horse.

Further rechecks were performed at 4 and 8 months postoperatively. On both occasions, the gelding did not show any signs of ataxia. The focal atrophy of the supraspinatus muscle remained unchanged. Mobility of the neck was slightly decreased towards the left, but palpation was unremarkable. Follow‐up radiographs were taken (Figure 8). The fracture line was no longer visible, and the implants were unchanged. It was recommended that the owner increase exercise and pursue physical therapy to address the focal muscle atrophy. At the time of manuscript submission, one year after surgery, the gelding was back in training, exercised in all gaits under saddle, showed no neurologic deficits, and performed to the rider's complete satisfaction.

## 4 DISCUSSION

This is the first report of a successful repair of a C6 AP fracture using lag screw fixation with the aid of CAS. The use of CAS provided significant advantages in both preoperative planning and intraoperative accuracy, making surgery of this complexity possible in the first place. Considering the initial positive evolution of the case presented here and given the limited information available regarding the surgical management of AP fractures in horses, it can be argued that continued conservative management and a wait‐and‐see approach may have resulted in the same outcome. However, the concerns of imminent spinal cord compression or foraminal stenosis due to exuberant callus formation and the possibility of a controlled, navigated direct repair favoring primary fracture healing were decisive for the owner to opt for surgical stabilization of the AP fracture in this case.

Lag screw fixation of minimally displaced fractures can be achieved through less invasive approaches compared to what is needed for conventional open reduction and internal fixation using plates or other implants. However, achieving accurate screw placement in the narrow and anatomically complex osseous structure is challenging.^3^ Historically, managing AP fractures has been limited to a conservative approach or ventral stabilization techniques.^3^ Intervertebral body fusion comes with risks as several delicate structures are located within close proximity to the surgical field.^12^ Thus, fatal complications such as injury of the spinal cord during drilling or laryngospasm due to excessive pressure on the vagal nerve have been reported, as well as catastrophic fractures of the adjacent vertebral body.^1^, ^12^, ^13^

With the availability of CBCT‐based CAS for navigated surgery and repeated intraoperative imaging, if needed, lag screw fixation was deemed a reasonably safe option to stabilize the fracture and minimize the risks of excessive callus formation. While an anatomically safe corridor could also be established with a larger open approach to expose the AP joint, we are skeptical that conventional intraoperative CT‐image guidance would allow surgeons to confidently and consistently achieve the required accuracy and control over this challenging drilling procedure. Moreover, using C‐clamp aiming devices to assist CT‐guided drilling nearly seems impossible in the cervical region of the horse. Alternatively, three dimensional (3D)‐printed drill guides could be used based on preoperative CT images of the affected vertebra. However, these require direct contact with a large bone surface, necessitating a more invasive and larger open approach. In the here described case, CAS allowed for precise preoperative planning, including the identification of optimal screw entry points, trajectory, and depth, and provided the surgeon with real‐time intraoperative orientation and control over the penetration depth of the drill bit. The intraoperative CBCT performed after screw placement revealed that the alignment, as well as screw position and length, were appropriate. However, it was not possible to assess the reduction of the fracture gap due to the beam hardening artifacts created by the screws. As the fracture was already eight weeks old by the time of surgery, a visually perceptible reduction was not expected to begin with.

The multiplanar reconstruction of the fracture site enabled the surgeon to virtually assess implant positioning, minimizing the risk of cortical breach or injury to adjacent structures. CAS is routinely used in spinal surgery in humans, for example, for the placement of pedicle screws. For this particular indication, CAS has been shown to have a higher placement accuracy than the traditional procedure and to reduce the risk of accidental perforation of the pedicle.^10^, ^14^ Furthermore, radiation exposure for the patient and the surgical team can be significantly reduced using a navigation system, negating the need for several control scans.^10^ However, reports on the use of CAS for surgical intervention on the equine spine are still lacking.

Ataxia was observed after surgery, which may have been caused by swelling due to edema or focal hemorrhage within the soft tissues surrounding the spinal cord induced by the surgical manipulations. Less likely, it could also have been caused by the residual effects of the suspected brain edema. Although initially severe, ataxia was only transient. With more experience, the procedure can be refined and expedited. This will help minimize trauma to the surrounding tissues and result in less compression of the spinal cord, thus reducing the occurrence of neurological deficits after surgery.

The suspected brain edema may have resulted from increased intracranial pressure (ICP) due to impaired venous drainage related to the horse's positioning. The increased ICP could have led to depression of the respiratory centers, thereby diminishing the respiratory drive. The subsequent accumulation of carbon dioxide may have further exacerbated the clinical signs observed during recovery. Notably, the suspicion of elevated ICP was based solely on clinical presentation and the rapid improvement following intravenous administration of hypertonic saline, rather than on direct or objective measurements. While this response supports the hypothesis of increased ICP, alternative explanations for the prolonged apnea must also be considered. Upper airway obstruction was considered unlikely due to the absence of clinical evidence such as abdominal breathing, abnormal thoracic expansion during manual ventilation, or unusual respiratory sounds. Residual anesthetic effects could have been present; however, a standard anesthetic protocol was used, usually linked with rapid postoperative respiratory recovery. Furthermore, there were no clinical signs of severe hypercapnia, severe hypothermia, hypotension, or circulatory collapse.

The triceps myopathy, most likely a compartment syndrome, observed after surgery was also thought to be induced by the horse's positioning, with the shoulder on the edge of the unpadded carbon fiber table. This would also explain the atrophy of the supraspinatus muscle resulting from compression of the suprascapular nerve. More careful positioning of the horse might have helped avoid these complications and needs to be improved in future cases.

While follow‐up radiographs did not reveal excessive callus formation, a follow‐up CT examination to prove primary fracture healing was not performed. This is an important limitation of the present case report. An additional general anesthesia would have been necessary to perform a follow‐up CBCT of diagnostic quality that allows full assessment of the quality of fracture healing. This was deemed unnecessary since the horse showed a highly satisfactory clinical evolution and was back in full training without any restrictions one year after surgery.

Overall, lag screw fixation of the AP fracture was considered successful, despite the perioperative complications. The application of CAS greatly facilitated the surgical approach and enabled precise screw placement without damaging surrounding neurovascular structures. The use of CAS for successful case management highlights its potential in equine cervical spine surgery. Lag screw fixation may be a viable alternative for the treatment of AP fractures in the future, but further research is needed to refine case selection and determine the long‐term outcome after the procedure.

## AUTHOR CONTRIBUTIONS

Käfer‐Karrer MJ, Med Vet: Participated in case management and prepared the first draft of the manuscript. De Preux M, Dr Med Vet, DECVS: Participated in case management and manuscript preparation. Van der Vekens E, Dr Med Vet, Dip ECVDI, PhD: Participated in case management and manuscript preparation. Mattei LI, Dr Med Vet: Participated in case management and manuscript preparation. Kuhlmann J, Dr Med Vet: Participated in case management and manuscript preparation. Klopfenstein Bregger MD, Dr Med Vet, DECVS: Participated in case management and manuscript preparation. Easley JT, DVM, DACVS (Large Animal): Provided advice on case management and manuscript preparation. Koch C, Dr Med Vet, DACVS (Large Animal), DECVS: Coordinated and participated in case management and manuscript preparation. All authors have read and approved the final version of the article.

## CONFLICT OF INTEREST STATEMENT

The authors declare no conflicts of interest related to this report.
